# Analysis of the Phenotypic Variability as Well as Impact of Early Diagnosis and Treatment in Six Affected Families With *ALDH7A1* Deficiency

**DOI:** 10.3389/fgene.2021.644447

**Published:** 2021-04-01

**Authors:** Xianru Jiao, Pan Gong, Ye Wu, Yuehua Zhang, Zhixian Yang

**Affiliations:** Department of Pediatrics, Peking University First Hospital, Beijing, China

**Keywords:** epilepsy, sibling, *ALDH7A1*, pyridoxine-dependent epilepsy, developmental delay

## Abstract

**Objective:**

To describe the clinical characteristics of 12 patients from six families with pyridoxine-dependent epilepsy (PDE) carrying *ALDH7A1* mutations, and analyze the impact of early diagnosis and treatment, as well as possible genotype–phenotype relationship.

**Methods:**

Clinical and genetics data of 12 patients were collected.

**Results:**

Family 1–3 presented with symptoms in the neonatal period, while family 4-6 presented during early infancy. In the same family, the age of onset was similar. The focal motor seizure appeared in all patients. The affected identical twins from family 4 were diagnosed with infantile spasms. Mutation analysis identified nine different *ALDH7A1* mutations among six families. The neurodevelopment of siblings in family 1 was mild delay and normal separately due to the minor difference of delayed diagnosis time. Siblings in family 2 showed severely delayed and normal development respectively due to the significant difference of a delayed diagnosis for 4 years. In family 5, although the difference of the delayed diagnosis time is up to 7 years, the nearly normal psychomotor development in both patients might be due to infrequent seizures before the delayed diagnosis. A severe phenotype exhibited in family 3, 4, and 6. The survived affected patients presented with severe developmental delay or refractory seizures and their twins or older sisters presented a similar clinical history and died in the early days of life. Mutation analysis showed D511N and IVS11 + 1G > A in family 3, V188A and exon1 deletion in family 4, and Y354C and exon 8–13 deletion in family 6.

**Conclusion:**

Patients from the same family often have the same phenotype, including onset age and seizure type. Early treatment with pyridoxine and infrequent seizures showed positive relationship with prognosis. The deletion of exon 1 and exon 8–13 might be associated with the severe phenotype.

## Introduction

Pyridoxine-dependent epilepsy (PDE, OMIM: 266100) is a clinical disorder that typically presents in infancy or early childhood, caused by a deficiency of aldehyde dehydrogenase 7 family member A1 (*ALDH7A1*) which was first identified in 2006 (Hunt et al., 1954; Mills et al., 2006). In 2016, the mutation in *PLPBP* was identified as another pathogenic gene for PDE (Darin et al., 2016). Whereas, according to a recent literature review, *PLPBP* associated disease was defined as *PLPBP* deficiency (OMIM: 617290), which is a vitamin B6-dependent disease (Wilson et al., 2019). The largest cohort of PDE patients reported to date in a single center was 33 cases, including two patients with *PLPBP* mutations (Jiao et al., 2020). The disease is characterized by intractable seizures which are unresponsive to antiepileptic drugs (AEDs), but can be controlled by pharmacologic doses of pyridoxine (Baxter, 2001). However, it is worth noting that even though pyridoxine supplementation succeeds in controlling clinical and electrical seizure activity, clinical outcome in PDE is usually poor, with 75% of treated patients experiencing developmental delay (Bok et al., 2012; van Karnebeek and Jaggumantri, 2015). Till now, over 300 cases of PDE have been identified since its first description in 1954 (Hunt et al., 1954; Jiao et al., 2020), although a majority of published mutations were reported in small case series or single case reports. So far, more than 180 pathogenic variants of *ALDH7A1* have been identified by systematic literature search (Coughlin et al., 2019; Jiao et al., 2020). However, due to the interference of other factors, it is difficult to analyze the phenotypes of patients with the same genotype, such as clinical characteristics, treatment, prognosis, etc., since most patients are uniquely affected individuals in the family.

The present study aims at describing 12 patients with PDE from six unrelated families. Of these, eight patients have previously been reported (family 1, 2, 3-I, 4-I, 5-I, and 6-I) (Jiao et al., 2020). It includes the initial clinical presentations, long-term follow-up and molecular data. The diagnosis of nine patients was confirmed by *ALDH7A1* sequencing, and the other three patients did not undergo genetic testing due to an early death, clinically diagnosed as PDE.

## Materials and Methods

### Ethics Statement

This study was approved by the Biomedical Research Ethical Committee of Peking University First Hospital. The individuals’ parents in this manuscript have given written informed consent to publish the case details.

### Patients

A retrospective chart review of 12 patients from six unrelated families were performed. Electroencephalogram (EEG), magnetic resonance imaging (MRI), biochemical studies, plasma amino acids and urine organic acids test were performed in nine patients. Neurodevelopmental assessment of family 3-I was performed according to Gesell intelligence scales, and other patients were evaluated through clinical judgment and parents’ questionnaires.

### Genetic Analysis

DNA extracted from peripheral blood from patients and other family members was analyzed using whole-exome sequencing (WES) with standard protocol (Yang et al., 2014). Sequence variants were checked with population databases gnomAD^1^ and the Combined Annotation-Dependent Depletion^2^ (CADD) score, and evaluated using Polyphen2, SIFT, and Mutation Taster. The variants were further confirmed by Sanger sequencing. The deletion of exons was validated by quantitative polymerase chain reaction (qPCR).

### Results

Genetic and clinical findings of the affected patients in six families were summarized in Table 1.

**TABLE 1:** Genetic and clinical findings of the affected patients in six families.

	**Family 1**		**Family 2**		**Family 3**		**Family 4**		**Family 5**		**Family 6**	
	**Family 1-I/Jiao et al. patient 6**	**Family 1-II/Jiao et al. patient 7**	**Family 2-I/Jiao et al. patient 27**	**Family 2-II/Jiao et al. patient 28**	**Family 3-I/Jiao et al. patient 3**	**Family 3-II**	**Family 4-I/Jiao et al. patient 29**	**Family 4-II**	**Family 3-I/Jiao et al. patient 31**	**Family 5-II**	**Family 6-I/Jiao et al. patient 20**	**Family 6-II**
Current age/Sex	9 y/F	2 y7 m/M	11 y/F	6 y/F	6 y/M	Died at 5 d	3 y6 m/F	Died at 4 m	8 y/M	6 m/M	7 y/F	Died at 8 m
Age at seizure onset	8 d	9 d	3 d	6 d	<24 h	3 d	4 m	2 m	5 m	4 m	2.5 m	/
Birth history	Meconium-stained Amniotic fluid	Normal	Normal	Normal	Asphyxia	/	Normal	Normal	Normal	Normal	Normal	/
Seizure type	FS, SE	FS	FS	FS	FS, SE	/	ES, FS	ES, FS	FS	FS	FS	/
Age at which B6 routine treatment started	10 m	9 d	4 y	6 d	10 m	NA	4 m3 d	NA	7 y	4 m	5 m	NA
B6 dose at the last follow-up	180 mg/day	40 mg/day	240 mg/day	160 mg/day	130 mg/day (7 mg/kg/day)	NA	40 mg/day	NA	150 mg/day	90 mg/day	210 mg/day	NA
	(7.8 mg/kg/day)	(3.3 mg/kg/day)	(1.7 mg/kg/day)	(3.2 mg/kg/day)			(4 mg/kg/day)		(3.3 mg/kg/day)	(9 mg/kg/day)	(10 mg/kg/day)	
Initial interictal EEG	Multifocal discharges	/	Generalized discharges	Abnormal	Left frontal and central discharges	/	Hypsarrhy- thmia	Hypsarrhy- thmia	Generalized discharges	/	Anterior discharges	/
EEG at last follow up	Normal	/	Normal	/	Abnormal	NA	Normal	NA	Centrotemporal spikes	/	Bilateral temporal and occipital discharges	NA
Brain MRI/Age	Normal/3 m	/	Normal/4 y	Normal/NA	CCH, PLVDM, SW/1 y9 m	/	NA	/	Normal/NA	/	Normal/NA	/
Psychomotor development	Mild delay	Normal	Severe delay	Normal	Severe delay	NA	Severe delay	NA	Normal	Normal	Moderate delay	NA
*ALDH7A1* (NM_001182.4)	c.965C > T	c.965C > T	c.1061A > G	c.1061A > G	c.1531G > A (p.D511N);	NA	c.563T > C (p.V188A);	NA	c.1061A > G	c.1061A > G	c.1061A > G (p.Y354C);	NA
	(p.A322V);	(p.A322V);	(p.Y354C);	(p.Y354C);	c.1008 + 1G > A(IVS11 + 1G > A)		Exon 1 deletion		(p.Y354C);	(p.Y354C);	Exon 8–13 deletion	
	c.952G > C	c.952G > C	c.1061A > G	c.1061A > G					c.364C > T	c.364C > T		
	(p.A318P)	(p.A318P)	(p.Y354C)	(p.Y354C)					(p.R122W)	(p.R122W)		

*FS, focal seizure; ES, epileptic spasms; SE, status epilepticus; NA, not available; /, not used or recorded; d, day; m, month; y, year; EEG, electroencephalogram; MRI, magnetic resonance imaging; SW, subarachnoid widened; PLVDM, posterior horn of lateral ventricle delayed myelination; CCH, corpus callosum hypoplasia.*

#### Family 1: family 1-I and family 1-II

##### Family 1-I

A 9-year-old female was the first child born to non-consanguineous Chinese parents. The mother suffered from hyperthyroidism during pregnancy, and the condition could be well controlled by taking propylthiouracil tablets. The patient had normal thyroid function unaffected by her mother. Meconium-stained amniotic fluid was found at birth. Focal seizures started on the eighth day after birth, manifesting limbs shake, followed by eyes slanting to the left or gaze fixed, purple lips, hypersalivation and screaming, which resolved rapidly after phenobarbital (PB) and diazepam infusion. Seizures reoccurred after the drugs withdrawn for a few days. Sometimes one seizure episode lasted more than half an hour, or occurred frequently within a few hours. At the 3 months after birth, valproic acid (VPA) treatment started, followed by topiramate (TPM), levetiracetam (LEV), clonazepam (CZP), carbamazepine, without significant effect. At the age of 6 months, oral pyridoxine (30 mg/day) was administered randomly and intermittently, but the relationship between every treatment period of pyridoxine and seizure condition was not clear. At the age of 10 months, pyridoxine (90 mg/day) was administered orally in combination with a variety of AEDs, and seizures could be controlled after a few days. Then, the diagnosis of PDE was suspected and confirmed by *ALDH7A1* genetic testing. All the AEDs were phased out and only oral pyridoxine (180 mg/day) maintained with seizure free since then. Brain MRI showed normal. Several EEG examinations all showed a small amount of multifocal discharges before high doses of pyridoxine treatment, returning to the normal after treatment. She showed mild motor developmental delay and intellectual disability at the age of 9 (the last follow-up).

##### Family 1-II

A boy is the younger brother of family 1-I. He was delivered at full term without any complications. He exhibited the first symptom at the ages of 9 days, presenting as unilateral or bilateral limbs clonus and eyes slanting to one side, accompanied by crying. The same condition occurred four times within 20 days. The patient was given oral pyridoxine (40 mg/day) at the onset of limbs clonus each time experimental, and the condition could be soon relieved. After the fourth episode, the patient was given 10 mg of pyridoxine orally every other day with unclear purpose, with no recurrent seizure observed. Then, the diagnosis was confirmed by the genetic test at 2 months after birth, and the dose of pyridoxine was increased to 40 mg/day without seizure relapse. Development was normal at the age of 2 years and 7 months (the last follow-up).

#### Family 2: Family 2-I and Family 2-II

##### Family 2-I

The female patient was the third child of non-consanguineous parents. The reason for the first two elective abortions was unknown. The girl was born after a normal pregnancy and uneventful delivery. Seizure started at the age of 3 days. The symptoms included loss of consciousness, squinting of the eyes, limbs stiffness with cyanotic lips, lasting for 2–3 minutes. The seizures were controlled by transient venous transfusion including pyridoxine on the same day. After one week, seizures relapsed, and were controlled once again by the above method. After that, seizures occurred 2–3 times per week, and was controlled for one week by the same method in hospital each time, with repeated onset within 3 years. However, oral pyridoxine was provided only at its low dose as a continuous nutrient supply continuously. The seizures could not be controlled by the combination therapy of LEV and TPM. At the age of 4, by revisiting the treatment history, routine oral pyridoxine (20 mg/day) therapy combined with valpromide was initiated. Since then, the seizure was controlled for 4 years. In the meantime, the seizures recurred after pyridoxine withdrawal for 1 month, and were controlled after re-administration. After being diagnosed with a genetic test at age nine, valpromide was gradually withdrawn and only oral pyridoxine (240 mg/day) maintained with seizure free since. EEG showed generalized discharges at 4 years old before oral pyridoxine therapy and then became normal after treatment at the age of 9. Brain MRI was normal. She had severe intellectual delay with normal movement development at the age of 11 (the last follow-up).

##### Family 2-II

A 6-year-old girl was 5 years younger than family 2-I. Seizure started at the age of 6 days, 3 days later than her elder sister. Because of the sister’s precedent, the seizures of family 2-II were controlled by intravenous pyridoxine on the day of onset, by the therapy of oral pyridoxine (20 mg/day) combined with valpromide, as was the case with her sister (PDE diagnosis undone for both at this time). So far, the oral dose of pyridoxine was 160 mg per day, with two relapses due to withdrawal at the age of 3 and 4. Together with her 9-year-old sister, the patient was diagnosed with PDE by genetic testing at the ages of four. EEG showed abnormal but the details were not clear at the age of 4. Brain MRI showed normal. Development was normal at the age of 6 (the last follow-up).

#### Family 3: Family 3-I and Family 3-II

##### family 3-I

The proband was the second male child born at term to non-consanguineous parents following an uneventful pregnancy. One hour after birth he presented with respiratory distress, along with clonus of the superior limbs. After 12 days of treatment in the hospital, the seizures ceased, and the patient was treated with PB orally for one week after discharge. Three months later, he developed recurrent seizures. LEV, CZP, and VPA were introduced in addition to PB. Before the age of 9 months, the patient had been repeatedly hospitalized due to seizure recurrence and discharged again after seizure improvement, although it was unknown which therapy methods were effective. At 10 months old, EEG showed left frontal and central discharges, and he was re-hospitalized. During the hospitalization, seizures were controlled after 3 days of intravenous infusion of pyridoxine, and it remained under control for 20 days. After discharge, pyridoxine was taken orally (80 mg/day) and the gene was tested at the same time. Then, the diagnosis of PDE was confirmed. All the AEDs were gradually withdrawn and only oral pyridoxine (130 mg/day) maintained with seizure free. Brain MRI showed subarachnoid widened, posterior horn of lateral ventricle delayed myelination, and corpus callosum hypoplasia. The EEG was still abnormal at the last follow-up. He had severe development delay at the age of 6 (the last follow-up). He could understand simple instructions and speak a few words occasionally.

##### Family 3-II

Family history study revealed that the first female child had developed epilepsy 3 days after birth and died 2 days later without available treatment details.

#### Family 4: Family 4-I and Family 4-II

##### Family 4-I

The two patients in family 4 were identical twins. The proband and her twin’s sister were born from an uneventful pregnancy. Epileptic spasms started at the age of 4 months. The seizures occurred almost every day. The first drug used was adrenocorticotropic hormone (ACTH). The next day, prednisone was substituted due to ACTH out of stock, and both LEV and pyridoxine (30 mg/day) were taken orally at 4 months of age. After 3 days of combination, the seizure was controlled. Prednisone was stopped after 6 months, while LEV and pyridoxine were continued for 10 months until the genetic results were known. At the age of 1 year and 1 month, LEV was gradually withdrawn and pyridoxine was maintained (40 mg/day). EEG showed hypsarrhythmia, and isolated or clustered epileptic spasms were captured during EEG monitoring at 4 months old. After 10 months of pyridoxine treatment, the EEG was normal. She had near-normal motor development and severe intellectual development delay at the age of three and a half (the last follow-up). She could understand simple instructions and speak a few words occasionally.

##### Family 4-II

Family history study revealed that the twin’s sister had presented a similar clinical history. She was diagnosed with infantile spasms at 2 months of age and died at the age of 4 months. Unfortunately, she never had a chance to be treated with pyridoxine before death.

#### Family 5: Family 5-I and Family 5-II

##### Family 5-I

The male patient was the first child of non-consanguineous parents. Pregnancy, labor, and early infancy were normal. The first episode occurred at the age of 5 months, and twitching of the unilateral limbs was noted, lasting for approximately several minutes. Days later, one seizure was successfully recorded during EEG monitoring, and the patient was treated with LEV subsequently. Astonishingly, similar seizures reoccurred during fever 2 years later, and were controlled again by increasing dose of LEV until the age of two and a half. Since then, the seizures recurred once almost every 2–3 years under treatment of LEV. At the age of 7, the diagnosis of PDE was confirmed by genetic testing, and treatment with pyridoxine was added (150 mg/day). After 1 year of treatment with pyridoxine, LEV was gradually withdrawn by an attempt. Several EEGs all showed generalized discharges before oral pyridoxine therapy and then showed functional centrotemporal spikes after pyridoxine treatment for half a year. Brain MRI was normal. Development was normal at the age of 8 (the last follow-up).

##### Family 5-II

The male child was born at term 8 years after his affected brother. At 4 months of life, the child was admitted to the hospital for seizures. He was immediately given pyridoxine therapy at dose of 90 mg per day from seizure onset due to the definite PDE diagnosis of his brother, and no recurrent seizures were observed. EEG and brain MRI were not performed. At the last follow-up, the development was normal (only 6 months old).

#### Family 6: Family 6-I and Family 6-II

##### Family 6-I

The proband was the second female child born at term to non-consanguineous parents following an uneventful pregnancy. The first seizures occurred at the age of two and a half months, manifesting eyes slanting to the left, brief twitches of right corner of the mouth and the unilateral limbs, followed by generalized convulsions, which lasted for approximately 20 min and resolved after diazepam infusion. Then, the patient had been hospitalized time after time during the next three episodes. The interval between each episode was about one week. Since pyridoxine was contained in the ingredients of each hospitalization infusion, treatment with oral pyridoxine at dose of 20 mg/day was started at the age of 5 months, in combination with oxcarbazepine. The seizure was not well controlled. At 3 years of age, PDE was confirmed by genetic testing and the oral dose of pyridoxine increased to 360 mg/day which still failed to control the seizures. At the last follow up (7 years old), pyridoxine (210 mg/day) with a combination of three AEDs could not bring the onset under control, only leading to a reduced frequency. EEG examination showed anterior discharges at the age of 4 years and 3 months. One year later, EEG showed bilateral temporal and occipital discharges. Brain MRI showed normal. At the last follow-up, the development was moderately delayed (7 years old).

##### Family 6-II

Family history study revealed that the first female child had presented a similar clinical history and died at 8 months of life without available treatment details.

### *ALDH7A1* Mutation Analysis

Molecular variants of *ALDH7A1* identified in six affected sibling pairs were summarized in Table 2. Mutation analysis identified nine different *ALDH7A1* mutations (NM_001182.4) among our six families, including six missense mutations: c.364C > T(p.R122W) in exon 4, c.563T > C(p.V188A) in exon 6, c.952G > C(p.A318P) in exon 11, c.965C > T(p.A322V) in exon 11, c.1061A > G(p.Y354C) in exon 12, c.1531G > A(p.D511N) in exon 17, one splice site mutation: c.1008 + 1G > A(IVS11 + 1G > A) and two deletion mutations: exon 1 deletion (c.(?_1-1)_c.(192 + 1_193-1) del) and exon 8–13 deletion (c.(681 + 1_696-1)_(c.1200 + 1_1201-1) del). Family 6-I was initially identified as homozygous mutations, with one paternal mutation site. Subsequently, the maternal deletion of exons was validated by qPCR: exon 8–13. In addition, Family 4-I underwent qPCR at the initial genetic testing, with one paternal missense mutation and one heterozygous deletion in exon 1, while her mother was still wild type.

**TABLE 2:** Details of molecular variants of *ALDH7A1* (NM_001182.4) identified in six affected sibling pairs.

**Mutation site**	**Pathogenicity prediction**			**CADD score**	**Frequency in the general population**
	**Polyphen2**	**SIFT**	**Mutation Taster**		
c.364C > T(p.R122W)	Probably damaging	Deleterious	Disease_causing	14.810	0.00003994
c.563T > C(p.V188A)	Probably damaging	Deleterious	Disease_causing	18.230	−
c.952G > C(p.A318P)	Possibly damaging	Tolerated	Disease_causing	18.080	−
c.965C > T(p.A322V)	Possibly damaging	Deleterious	Disease_causing	21.700	0.00069
c.1061A > G(p.Y354C)	Probably damaging	Deleterious	Disease_causing	19.690	0.0000544
c.1531G > A(p.D511N)	Probably damaging	Deleterious	Disease_causing	21.000	−
c.1008 + 1G > A(IVS11 + 1G > A)	−	−	Disease_causing	14.040	0.000024
Exon 1 deletion	−	−	−	−	−
Exon 8–13 deletion	−	−	−	−	−

## Discussion

The relationship between genotype and phenotype in monogenic diseases is important to predict the onset, severity of diseases, and prognosis. However, previous studies have confirmed that the relationship between phenotype and genotype of PDE was highly complex (Mills et al., 2010). In addition to genotype, delayed diagnosis, age of onset, and drug maintenance dose could also affect phenotype (Mills et al., 2010; Jiao et al., 2020). Here, by analyzing differences in severity within the same family or among different families, we revealed phenotypic variability, impact of early diagnosis and treatment on prognosis.

In the present case series, family 1–3 presented with symptoms in the early neonatal period, while family 4–6 presented during early infancy. In the same family, the age of onset was similar. Among various seizure types, the focal seizure appeared in all patients. In agreement with these data, previous studies have also concluded that there were various forms of seizures, and focal seizures was the most common (30%) (Basura et al., 2009). Besides, the other two patients (family 1-I and family 3-I) manifested as recurrent focal seizures, with the occurrence of status epilepticus. Family 1-II did not show status epilepticus due to early diagnosis and treatment with pyridoxine. In family 4-I, the onset was mainly characterized by epileptic spasm with hypsarrhythmia on EEG, and family 4-II, the twin’s sister of family 4-I, was similar. By describing five patients from three unrelated families, Marguet et al. (2016) also described similar clinical manifestation in the same lineage. Therefore, patients from the same family often have the same phenotype including onset age and seizure type. Of the cases for which clinical data were available, 14% (27/188) of patients with *ALDH7A1* deficiency had a phenotype consistent with infantile spasms (Basura et al., 2009; Bennett et al., 2009; Mills et al., 2010; Scharer et al., 2010; Pérez et al., 2013; Tlili et al., 2013; Mefford et al., 2015; van Karnebeek et al., 2016; Al Teneiji et al., 2017; Falsaperla et al., 2018; Jiao et al., 2020), as in our cohort of family 4. Although infantile spasm accounts for relatively few cases in patients with PDE, it should still be considered to try high-dose pyridoxine treatment for such patients in order to timely diagnosis and treatment, and obtain a good prognosis.

Though both patients from family 5 were well controlled and had a good prognosis, the initial diagnosis of PDE in family 5-I was uncertain due to a favorable response to LEV monotherapy. The mutations carried by family 5-I were Y354C and R122W. The pathogenicity of Y354C has been reported before (Nam et al., 2012; Xue et al., 2015). So, the pathogenicity of R122W has not been very clear. Whereas, since the diagnosis of PDE could not be excluded, pyridoxine was added. Thus, LEV and pyridoxine were maintained at the same time after genetic testing for one year and a half. However, after the onset of the younger brother (family 5-II), the similar onset age with the older brother and prompt response to pyridoxine monotherapy confirmed the diagnosis of PDE. At this point, the LEV treatment of family 5-I was withdrawn, and no recurrence was found after that, which also further proved the pathogenicity of R122W. The above difficulties in diagnosing PDE have also been reported in previous studies (Coker, 1992; Baxter, 1999). Therefore, the phenotype of family 5-I extended the phenotype of PDE and again suggested that PDE should not be excluded by the short-term or even long-term treatment response of AEDs.

In the previous report, neurodevelopmental outcome was impaired in the majority of PDE cases (Bok et al., 2012; van Karnebeek and Jaggumantri, 2015). Studies had shown that development was dependent on an early diagnosis and initiation of treatment in the first year of life (Coughlin et al., 2015; Al Teneiji et al., 2017). However, Mills et al. (2010) proved that it was difficult to draw conclusions regarding the relationship of outcome to age at diagnosis and treatment with pyridoxine. Moreover, as reported by Rankin et al. (2007), there was no differential effect on cognitive outcome between prenatal and postnatal pyridoxine treatment, with all three siblings having moderate to severe learning disability. In our study, in family 2, although the genotype, age of onset, and seizures type of the two patients were the same, the family 2-II who started treatment with pyridoxine on the day of the first episode had normal neurodevelopment. On the contrary, for family 2-I, the diagnosis of PDE and regular pyridoxine therapy was delayed for 4 years and the neurodevelopment was severely delayed. Furthermore, due to the minor difference in delayed diagnosis time, the neurodevelopment of the two patients in family 1 was mildly delayed and normal, separately. For the other condition, it was delayed for up to 7 years for the family 5-I to be diagnosed as PDE and treated by pyridoxine, whereas family 5-II was immediately diagnosed and treated. However, both patients had nearly normal neurodevelopment. The reason might be due to infrequent seizures and well treatment response to LEV of family 5-I. In the case of a sibling reported by Yeghiazaryan et al. (2011), the elder sister started pyridoxine treatment 10 days after the initial seizure, but did not became seizure-free. In contrast, the younger brother began pyridoxine treatment after the first seizure and became seizure-free. In addition, the second affected female patient who was immediately given high dose pyridoxine therapy from seizure onset showed milder developmental delay than her elder affected sister who had delayed treatment (Marguet et al., 2016). Therefore, by comparing different patients in the same family, we concluded that early treatment with pyridoxine or relatively infrequent seizures was associated with well prognosis, although the development might not be completely normal.

In three families with affected children who died early, the phenotype of surviving patients was more severe who showed severe developmental delay, abnormal birth history, or refractory seizures: in family 3, the second affected child (family 3-I) showed an abnormal birth history and severe developmental delay; family 4-I still had severe developmental delay even though the seizure was timely controlled in the early stage; in particular, family 6-I who carried a deletion of exon 8–13 still had seizures even with high dose of pyridoxine combined with multiple AEDs and showed moderate developmental delay. These might be due to the fact that the mutations themselves had a greater impact on function. For family 4-I and family 6-I, according to their mutation site, the deletions of exon 8–13 and exon 1 might had a greater impact on function due to the absence of a large segment of the gene involved in *ALDH7A1*, resulting in serious gene function loss. For family 3-I, IVS11 + 1G > A was first reported by us, and previous study identified that this site was high prevalence in Chinese PDE patients (Yang et al., 2014; Xue et al., 2015; Jiao et al., 2020). Moreover, the corresponding phenotype did not indicate a severe phenotype or early death (Yang et al., 2014; Xue et al., 2015; Jiao et al., 2020). So, his severe phenotype might be attributed to D511N. However, the mutant site D511N only appeared in our cohort, so we could not compare the phenotype to patients with the same genotype (Jiao et al., 2020). Therefore, the mutations D511N might be associated with the severe phenotype, but more cases are needed to verify it.

## Conclusion

This study involved 12 patients with PDE from six unrelated families. By analyzing differences in severity within the same family or among different families, we revealed that patients from the same family often have the same phenotype including onset age and seizure type. In additional, early treatment with pyridoxine and infrequent seizures showed positive relationship with prognosis. The deletion of exon 1 and exon 8–13 might be associated with the severe phenotype.

### LIMITATIONS

Our study had several limitations. At the time of retrospective collection of medical records, the dose of intravenous pyridoxine for transient therapy and the time interval between recurrence were ambiguous in some patients before diagnosis of PDE. There is no standard dose of pyridoxine treatment for each patient. In addition, three patients who died prematurely were diagnosed with PDE only on a clinical level, rather than passing a genetic test. However, by describing six pairs of affected siblings, this study focuses on the analysis of phenotypes, diagnosis, treatment and prognosis, which is significant for the diagnosis and guidance of PDE treatment.

## Data Availability Statement

The original contributions presented in the study are included in the article/Supplementary Material, further inquiries can be directed to the corresponding author/s.

## Ethics Statement

The studies involving human participants were reviewed and approved by Biomedical Research Ethical Committee of Peking University First Hospital. Written informed consent to participate in this study was provided by the participants’ legal guardian/next of kin.

## Author Contributions

ZY conceptualized and designed the study, coordinated the study overall, and revised the manuscript. XJ co-designed the study, drafted the initial manuscript, and revised the manuscript. PG helped to collect and summarize data and revised the manuscript. YW and YZ interpreted data and critically reviewed the manuscript. All authors approval of the final revision of the article.

## Conflict of Interest

The authors declare that the research was conducted in the absence of any commercial or financial relationships that could be construed as a potential conflict of interest.

## Footnotes

1 http://gnomad.broadinstitute.org/

2 https://cadd.gs.washington.edu/
